# A Practical Guide to the Evaluation of Compensation Strategies for Gait Impairment in Parkinson’s Disease

**DOI:** 10.3233/JPD-223296

**Published:** 2022-09-02

**Authors:** Anouk Tosserams, Jorik Nonnekes

**Affiliations:** a Center of Expertise for Parkinson & Movement Disorders, Department of Neurology, Donders Institute for Brain, Cognition and Behaviour, Radboud University Medical Centre, Nijmegen, The Netherlands; b Center of Expertise for Parkinson & Movement Disorders, Department of Rehabilitation, Donders Institute for Brain, Cognition and Behaviour, Radboud University Medical Centre, Nijmegen, The Netherlands; c Department of Rehabilitation, Sint Maartenskliniek, Nijmegen, The Netherlands

**Keywords:** Parkinson’s disease, gait, rehabilitation, physical therapy

## Abstract

The application of compensation strategies is an important element of gait rehabilitation in persons with Parkinson’s disease. While the efficacy of these strategies is generally very high, a tailored, personalized approach is necessary to determine the optimal strategies for every patient. We propose a straightforward, practical guide to the evaluation of compensation strategies in clinical practice: considering the primary gait target(s) to optimize functional mobility, the context in which the strategies will be employed in daily life, and patient-specific personal preferences. Furthermore, we introduce www.walkingwithparkinson.com as a resource on the available compensation strategies, developed specifically for persons with Parkinson’s disease, their carers, and (allied) healthcare professionals.

Gait impairments are among the most common and disabling symptoms of Parkinson’s disease (PD). Management consist of complementary pharmacological and non-pharmacological treatment options [1]. Physiotherapy is a cornerstone of the non-pharmacological pillar, and the application of compensation strategies is one of the elements of physiotherapy. These strategies comprise a wide range of ‘detours’ to improve walking capacity (Table 1) [2]. While the efficacy of gait compensation strategies is generally very high, the effects of specific strategies vary greatly between patients: what works spectacularly well in one patient, has no effect—or even aggravates gait impairment—in the next [3]. Therefore, a personalized approach to gait rehabilitation is imperative to find a suitable strategy for every person with PD and gait impairment. Ideally, every person with PD and gait impairment should be informed about compensation strategies by a healthcare provider (for example, by a skilled PD physiotherapist), who can also provide expert guidance during their search for the most appropriate strategies. To this end, a variety of strategies should be systematically evaluated in a trial-and-error manner, to identify which suit the patient’s unique situation and needs best. However, a study in 320 Dutch physiotherapists and other PD healthcare professionals revealed that 87% did not use such systematic approach, reportedly due to limited knowledge and skills on the topic [4].

**Table 1:** Compensation strategies for gait impairment in Parkinson’s disease

Category	Suspected principle mechanism	Examples of strategies
1. External cueing	–Introduction of goal-directed behavior by introducing a movement reference or target;	–Walking to the rhythm of music;
		–Stepping over lines on the floor;
		–Bouncing a ball.
	–Assist in filtering and prioritizing tasks, especially during response selection under conflict.
2. Internal cueing	–Helps to achieve focused attention toward specific components of gait, to shift from automatic to goal-directed motor control.	–Counting;
		–Focusing on a specific component of the gait cycle (e.g., making a heel strike).
3. Changing the balance requirements	–Facilitates the ability to make lateral weight shifts, thereby easing the swing phase of the unloaded leg, particularly in gait initiation or turning.	–Using walking aids;
		–Making a volitional weight shift before gait initiation;
		–Making wider turns.
4. Altering the mental state	–Enhancing general alertness and arousal. This may help shift from automatic to goal-directed motor control.	–Reducing anxiety (e.g., mindfulness);
		–Increasing motivation (e.g., encouraging oneself).
5. Action observation and motor imagery	–Activation of the mirror neuron system may facilitate cortically generated movement.	–Mimicking the gait pattern of another person.
6. Adopting a new walking pattern	–Using alternate motor programs that may be less overlearned and less dependent on the automatic mode of motor control.	–Skipping;
		–Walking backwards or;
		–Running;
		–Making skating movements.
7. Alternatives to walking^*^	–Walking difficulty may be a task-specific problem.	–Riding a bicycle;
		–Skateboarding;
		–Riding a scooter;
		–Roller skating.

This table was adopted from: Nonnekes et al. [2] Compensation strategies for gait impairments in Parkinson’s disease: a review. *JAMA Neurology*. ^*^ The use of these alternatives to walking should generally only be explored in patients that already have prior experience in using that mode of transportation, to avoid dangerous situations.

Here, we present a straightforward, practical guide specifically focused on the evaluation of compensation strategies for gait impairment in PD, in support of complementary pharmacological treatments and other elements of physiotherapy [5]. Using this stepwise approach - based on scientific evidence as well as our personal clinical expertise on the topic - we aim to provide healthcare professionals with the tools to evaluate the broad variety of compensation strategies in a systematic, tailored and achievable manner.

## 1. WHAT ELEMENT OF GAIT IS THE PRIMARY TARGET?

First, determine your primary gait target—of course, there may be multiple targets within one patient in order to optimize functional mobility. Different strategies likely affect different spatiotemporal gait parameters. For example, due to the nature of the cues, auditory cueing (e.g., using a metronome) likely targets stride time, whereas visual cueing (e.g., stepping over lines) targets stride length [6]. Therefore, depending on the primary gait target you and your patient wish to improve (e.g., alleviating episodes of freezing or festination, improving gait rhythmicity, increasing step length, increasing gait speed, improving posture, improving gait initiation) the choice of the most appropriate strategies varies. With progressing disease, the primary target(s) may shift due to increased disability or cognitive decline, which is why—ideally—strategies should be re-evaluated periodically.

## 2. IN WHAT CONTEXT WILL THE STRATEGY BE APPLIED IN DAILY LIFE?

Next, evaluate a strategy’s efficacy in the context in which it will most likely be applied in daily life. This is important because the efficacy of the strategies tends to vary depending on the context in which they are used [3]. The context could entail a certain environment (e.g., what works in the consulting room does not necessarily translate to a crowded marketplace), or a specific situation (e.g., involving an element of time-pressure, or during dual-tasks such as talking while walking). To this end, if possible, it is especially helpful to arrange a home visit to make an inventory of problems that need to be addressed. Perhaps a specifically problematic turn in the kitchen could be tackled by taping down lines on the floor to prevent the occurrence of freezing [7]. If home visits are unfeasible, you could ask your patient to bring a videotape of their home environment to the consultation, or even videotape themselves in the specific areas or situations in which they experience most difficulties.

## 3. DOES YOUR PATIENT HAVE ANY SPECIFIC PERSONAL PREFERENCES?

Lastly, it is important to consider your patient’s personal preferences. While wearing laser shoes or adopting a new walking pattern may be highly effective, some patients will prefer strategies that are not noticeable to bystanders, avoiding stigmatization or feelings of embarrassment [8]. In these cases, the search for appropriate strategies could be narrowed down to strategies like internal cueing, altering the mental state, action observation and motor imagery. Persons who like to walk alone will probably not be helped by applying action observation, and people who are hesitant to wear headphones in public (e.g., in traffic) will probably not want to use a metronome when walking outdoors. Importantly, you should consider your patient’s cognitive status and learnability, which may largely influence the feasibility of certain compensation strategies. In other words, besides being effective, a strategy should also be usable in daily life according to the intended user: your patient.

Another way to incorporate your patient’s preferences is by making use of their skills or hobbies. If they love music, try walking to their favorite tune over using a metronome. If they are, or used to be, an avid ice skater, have them try making skating motions instead of adopting their usual walking pattern. The search for appropriate strategies is truly a collaborative effort between you and your patient.

## HOW TO EDUCATE YOURSELF AND YOUR PATIENTS ON COMPENSATION STRATEGIES

Compensation strategies are often spontaneously ‘invented’ by persons with PD themselves. Consequently, many new strategies do not reach healthcare professionals or other patients, even though additional resources on the available strategies are in high demand. To meet this demand, we have developed a dedicated online platform (https://www.walkingwithparkinson.com) with information on compensation strategies, where patients and professionals can also inspire and learn from each other by sharing videos of their own strategies. The platform is currently available in English and Dutch.

## CONFLICT OF INTEREST

The authors have no conflict of interest to report.
